# Supplementary material for: Perturbations of Lipids and Oxidized Phospholipids in Lipoproteins of Patients with Postmenopausal Osteoporosis Evaluated by Asymmetrical Flow Field-Flow Fractionation and Nanoflow UHPLC–ESI–MS/MS
Source: Antioxidants (Basel). 2020 Jan 5;9(1):46. doi: 10.3390/antiox9010046 (PMC7022717; doi:10.3390/antiox9010046)
Supplement: Supplementary file 1 [file antioxidants-09-00046-s001.pdf]

Supplementary material for

Perturbations of lipids and oxidized phospholipids in lipoproteins of patients with postmenopausal osteoporosis by asymmetrical flow field-flow fractionation and nanoflow UHPLC-ESI-MS/MS

Kang Geun Lee, Gwang Bin Lee, Joon Seon Yang, and Myeong Hee Moon*

Department of Chemistry, Yonsei University, Seoul 03722, Korea

Table of Contents

Figure S1..S-2
Figure S2..S-3
Figure S3 ....S-4
Figure S4 ....S-5
Table S1.. S-6
Table S2..S-7
Table S3 ....S-8
Table S4 ....S-9
Table S5 ....S-18
Table S6 ....S-21

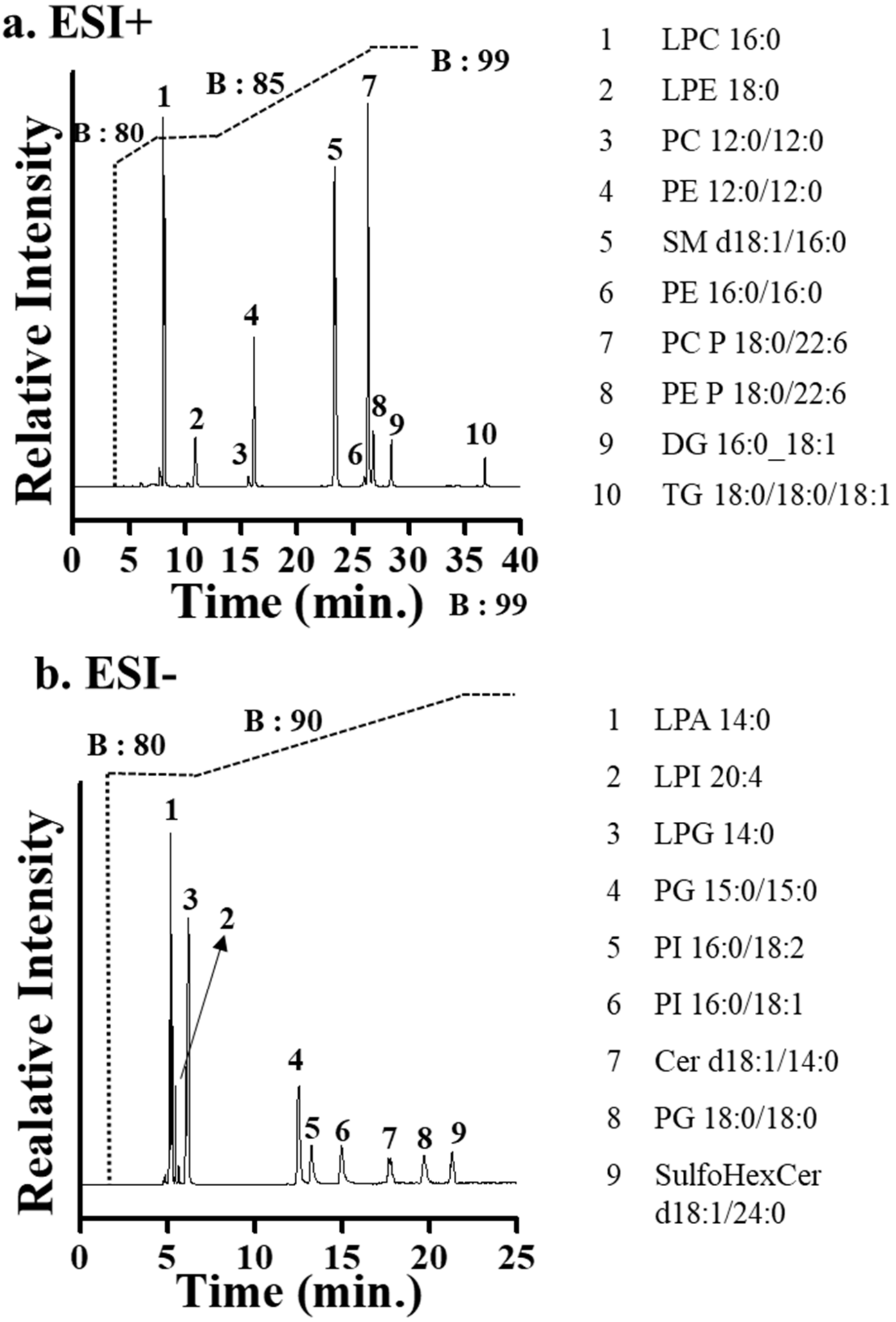

Figure S1. Base peak chromatograms of lipid standards in a) positive and b) negative ion mode of nUHPLC-ESI-MS/MS. Dotted lines represent the gradient elution condition represented with the increase of the composition of mobile phase B.

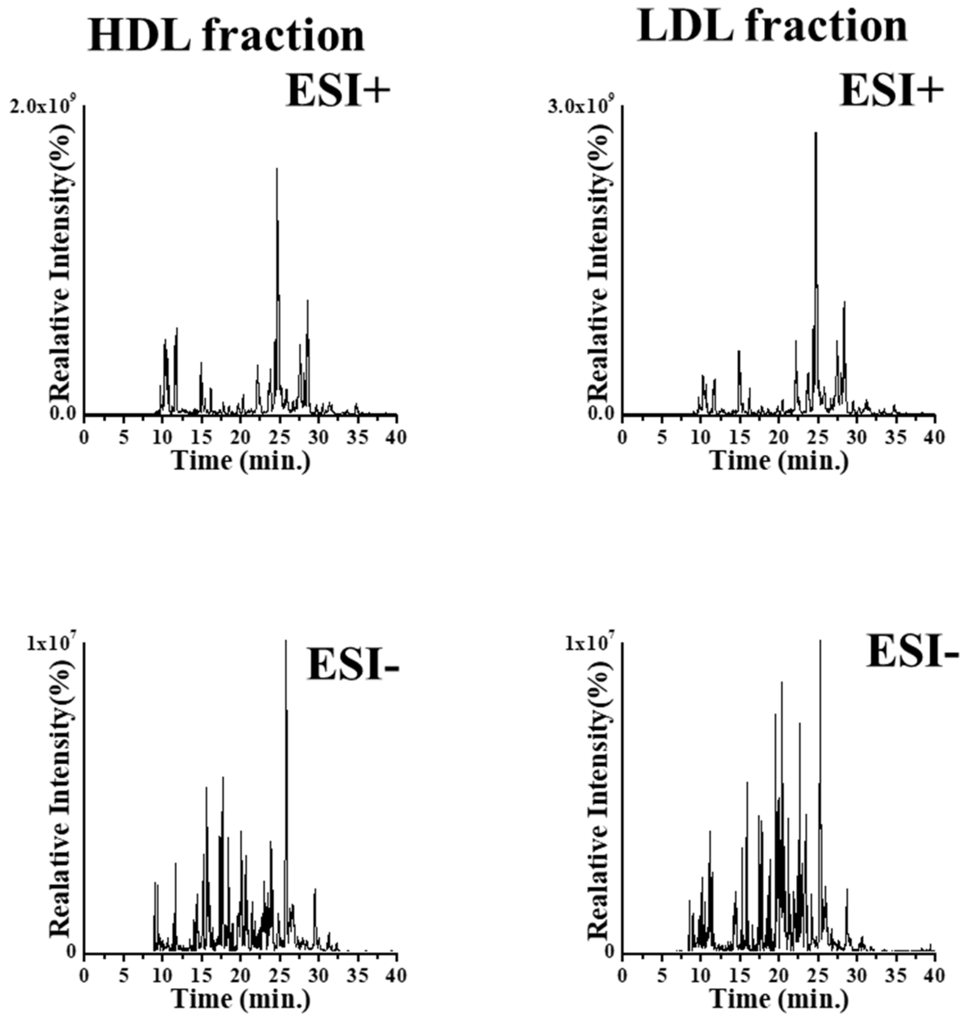

Figure S2. BPC's of lipid extracts from HDL and LDL fraction of a plasma sample pooled with patients (PMOp) and controls obtained by nUHPLC-ESI-MS/MS.

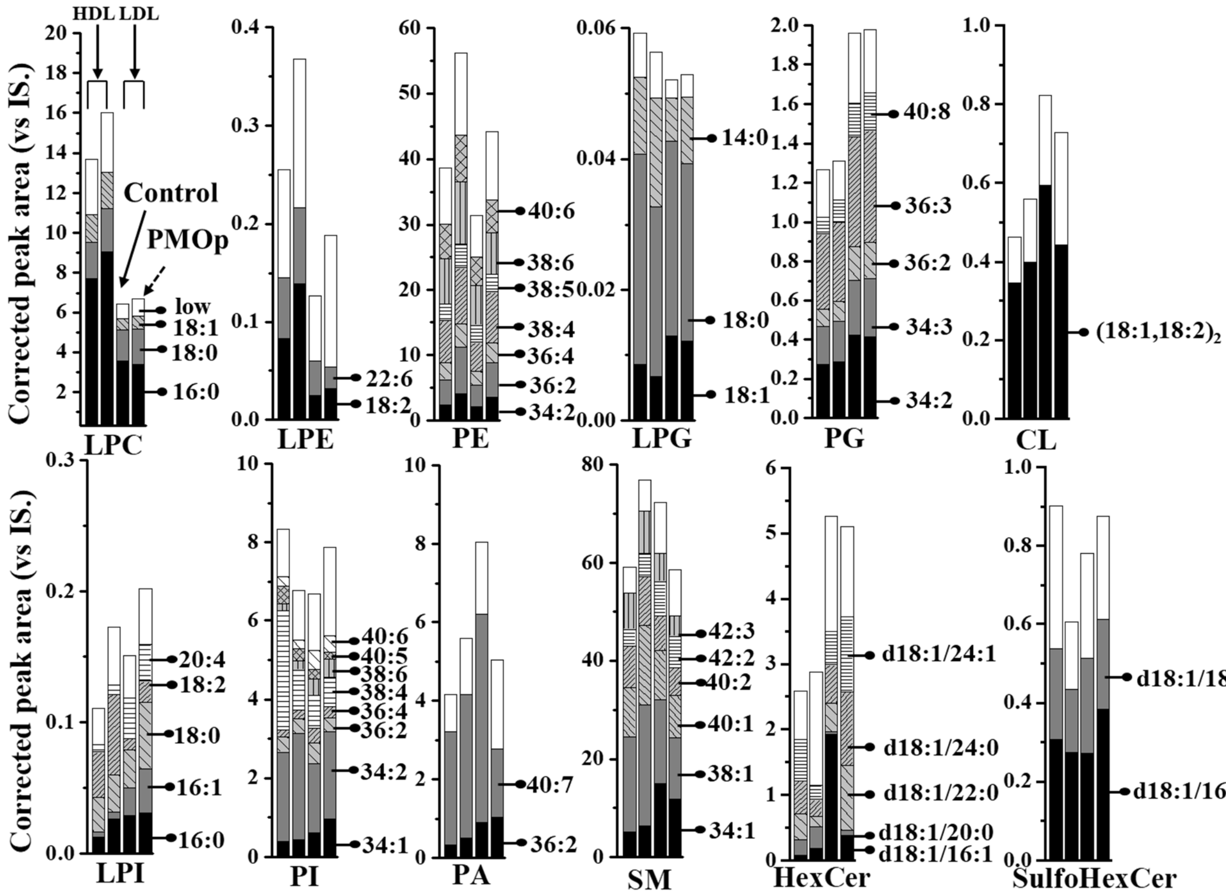

Figure S3. Stacked bar graphs of lipid species showing the summed amounts of (>1.5-folds and $p < 0.05$) in PMOp (n=10) groups in comparison to controls (n=10).

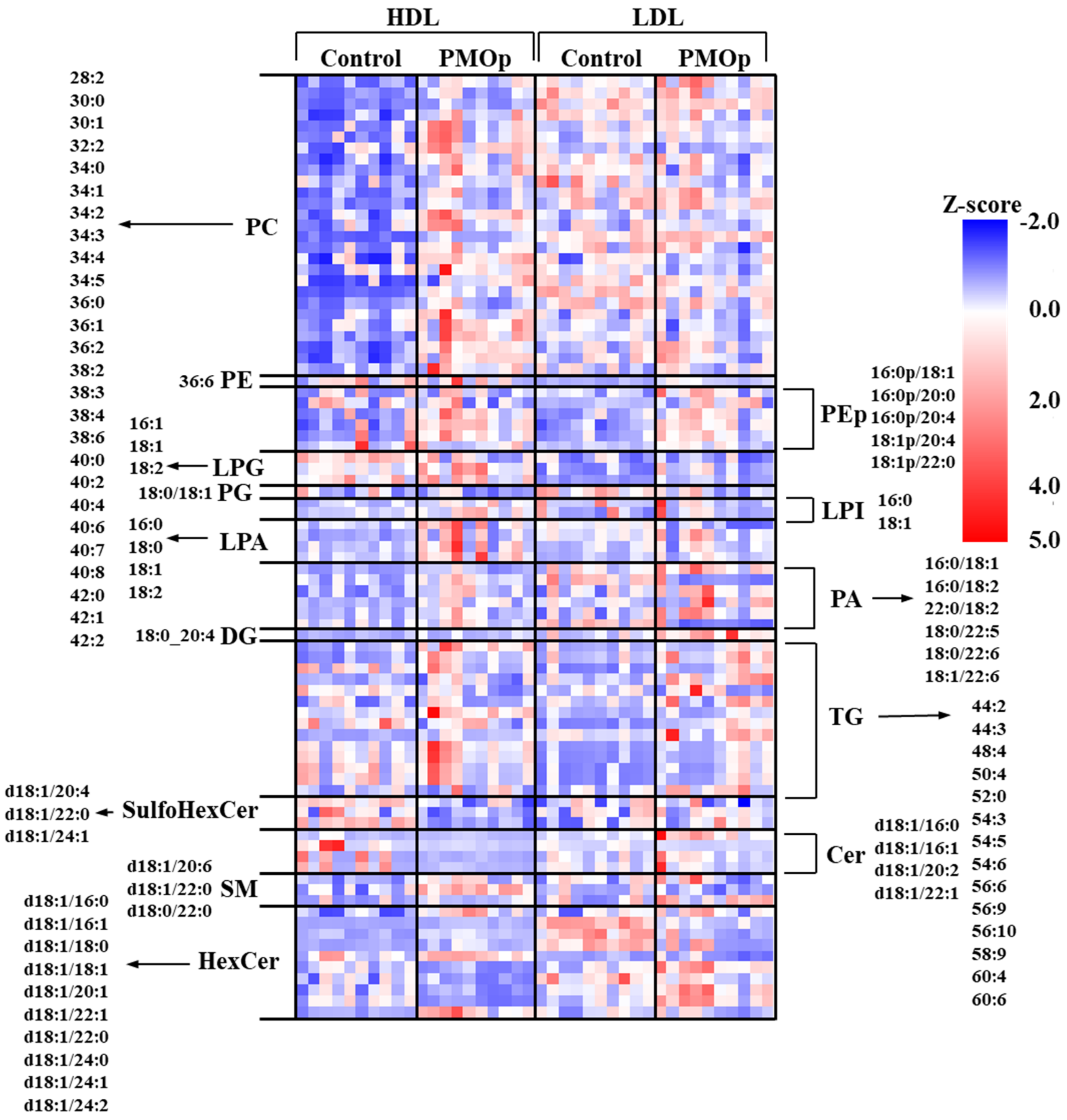

Figure S4. Heat map of selected lipid species showing the significant difference (>1.5-folds and $p < 0.05$) in PMOp (n=10) groups in comparison to controls (n=10).

Table S1. Demographic data for control and PMOp patients.

variables	control (n=10)	PMOp (n=10)
age (years)	72.1 ± 3.9	60.6 ± 7.9
height (cm)	150.8 ± 6.1	151.0 ± 5.2
weight (kg)	56.6 ± 7.1	51.8 ± 4.0
BMI (kg/m^2)	24.8 ± 1.7	22.7 ± 2.3

Abbreviations: PMOp : PostMenopausal Osteoporosis, BMI : Body Mass Index

Table S2. List of lipid internal standards and their precursor ion, product ion and collision energy used in SRM quantification process.

class	molecular species	precursor ion		product ion	collision energy (V)
		detected form	m/z		
LPC	17:0	$[M+H]^+$	510.5	$[Pcho+H]^+$	25
PC	13:0/13:0	$[M+H]^+$	650.5	$[Pcho+H]^+$	40
LPE	17:1	$[M+H]^+$	466.5	$[M+H-141]^+$	20
PE	17:0/17:0	$[M+H]^+$	720.5	$[M+H-141]^+$	20
LPA	17:0	$[M-H]^-$	423.5	$[RCOO]^-$	40
PA	17:0/17:0	$[M-H]^-$	675.5	$[RCOO]^-$	40
LPG	17:1	$[M-H]^-$	495.5	$[RCOO]^-$	35
PG	15:0/15:0	$[M-H]^-$	693.5	$[RCOO]^-$	35
LPI	17:1	$[M-H]^-$	583.5	$[RCOO]^-$	50
PI	12:0/13:0	$[M-H]^-$	711.5	$[RCOO]^-$	50
SulfoHexCer	d18:1/17:0	$[M+H]^+$	794.5	$[d18:1]^+$	40
SM	d18:1/17:0	$[M+H]^+$	717.5	$[Pcho+H]^+$	30
Cer	d18:1/17:0	$[M+H]^+$	552.5	$[d18:1]^+$ & $[d20:1]^+$	30
HexCer	d18:1/17:0	$[M+H]^+$	714.5	$[d18:1]^+$	40
CL	$(14:1)_3(15:1)$	$[M-H]^-$	622.5	$[M-H]^-$	40
DG	17:0/17:0 D_5	$[M+NH4]^+$	619.5	$[M+NH_4-RCOONH_4]^+$	20
TG	17:0/17:0/17:1 D_5	$[M+NH4]^+$	870.7	$[M+NH_4-RCOONH_4]^+$	25

* Pcho : phosphocholine

Table S3. Limit of detection (LOD) and limit of quantification (LOQ) of lipid standard lists spiked to each lipid extract.

class	molecular species	HDL			LDL		
		LOD(pmol)	LOQ(pmol)	R^2	LOD(pmol)	LOQ(pmol)	R^2
LPC	17:0	0.005	0.015	0.983	0.005	0.017	0.987
PC	13:0/13:0	0.003	0.009	0.985	0.003	0.010	0.995
LPE	17:1	0.023	0.075	0.993	0.025	0.083	0.985
PE	17:0/17:0	0.010	0.031	0.984	0.009	0.028	0.982
LPG	17:1	0.011	0.036	0.996	0.010	0.033	0.997
PG	15:0/15:0	0.017	0.054	0.993	0.015	0.049	0.972
LPI	17:1	0.020	0.067	0.993	0.018	0.061	0.993
LPA	17:0	0.006	0.017	0.998	0.005	0.015	0.995
PA	17:0/17:0	0.010	0.030	0.998	0.009	0.027	0.995
DG	17:0/17:0	0.007	0.024	0.996	0.008	0.027	1.000
TG	17:0/17:1/17:0	0.014	0.045	0.981	0.015	0.050	0.980
SM	d18:1/17:0	0.015	0.050	0.998	0.017	0.055	0.999
Sulfo HexCer	d18:1/17:0	0.013	0.041	0.997	0.014	0.046	0.997
Cer	d18:1/17:0	0.013	0.043	0.997	0.014	0.047	1.000
HexCer	d18:1/17:0	0.005	0.017	0.999	0.006	0.019	0.994
CL	$(14:0)_4$	0.041	0.135	0.992	0.045	0.150	0.995

Table S4. Corrected peak area value and fold ratio (PMOp/Control) of identified lipids in HDL and LDL fractions obtained by nUHPLC–ESI–MS/MS. Species emphasized with bold represents > 1.5-fold change and underlined numerals for high abundance in each lipid class. * for p-value < 0.05.

class	molecular species	*m/z*	HDL				LDL			
			control (n=10)	PMOp (n=10)	PMOp/control	abun. (%)	control (n=10)	PMOp (n=10)	PMOp/control	abun. (%)
LPC	14:0	468.5	0.24±0.02	0.30±0.02	1.25±0.15	1.74	0.01±0.00	0.02±0.00	**1.56±0.20**	0.23
(12)	16:0	496.5	7.71±0.76	9.06±0.42	1.17±0.13	56.10	3.58±0.18	3.38±0.09	0.94±0.05	55.46
	16:1	494.5	0.53±0.07	0.60±0.04	1.13±0.17	3.96	0.05±0.00	0.07±0.00	**1.53±0.15**	0.74
	18:0	524.5	1.81±0.20	2.14±0.08	1.19±0.14	13.05	1.52±0.07	1.77±0.06	1.16±0.06	24.07
	18:1	522.5	1.41±0.10	1.86±0.06	1.32±0.11	10.31	0.59±0.03	0.66±0.02	1.12±0.06	9.20
	20:2	548.5	0.02±0.00	0.03±0.00	**1.66±0.17**	0.12	0.02±0.00	0.02±0.00	1.15±0.12	0.24
	20:3	546.5	0.20±0.02	0.22±0.01	1.09±0.12	1.46	0.09±0.00	0.11±0.00	1.19±0.08	1.37
	20:4	544.5	0.78±0.08	0.86±0.05	1.10±0.12	5.77	0.24±0.01	0.30±0.01	1.24±0.08	3.86
	20:5	542.5	0.43±0.07	0.30±0.03	0.69±0.13	3.34	0.09±0.01	0.08±0.00	0.85±0.10	1.52
	22:5	570.5	0.11±0.01	0.10±0.01	0.92±0.09	0.76	0.05±0.00	0.05±0.00	1.08±0.09	0.72
	22:6	568.5	0.45±0.05	0.53±0.03	1.16±0.15	3.34	0.15±0.00	0.20±0.01	1.29±0.08	2.40
	24:1	606.5	0.01±0.00	0.01±0.00	1.02±0.18	0.04	0.01±0.00	0.01±0.00	0.85±0.07	0.19
PC	28:2	674.5	0.00±0.00	0.01±0.00	**3.41±0.40***	0.01	0.01±0.00	0.02±0.00	**1.56±0.13**	0.01
(28/48)	30:0	706.5	0.15±0.01	0.31±0.02	**2.08±0.21***	0.12	0.55±0.05	0.57±0.03	1.02±0.11	0.34
	32:0	734.5	0.88±0.05	1.70±0.15	**1.93±0.20***	0.73	4.48±0.25	4.57±0.25	1.02±0.08	2.75
	32:1	732.5	1.18±0.14	2.06±0.21	**1.74±0.27**	0.98	2.10±0.19	1.92±0.14	0.91±0.11	1.29
	32:2	730.5	0.17±0.02	0.37±0.03	**2.13±0.26***	0.14	0.42±0.04	0.37±0.01	0.87±0.08	0.26
	34:0	762.5	1.18±0.08	2.74±0.16	**2.32±0.21***	0.98	1.86±0.13	2.01±0.10	1.08±0.09	1.15
	34:1	760.5	12.14±0.88	27.61±1.08	**2.28±0.19***	10.04	18.12±1.66	19.27±0.96	1.06±0.11	11.15
	34:2	758.5	25.60±3.67	46.10±4.31	**1.80±0.31***	21.18	26.47±2.00	22.79±1.11	0.86±0.08	16.28
	34:3	756.5	1.18±0.11	2.15±0.21	**1.82±0.25***	0.97	2.16±0.17	1.99±0.12	0.92±0.09	1.32
	34:4	754.5	0.05±0.00	0.13±0.01	**2.31±0.24***	0.05	0.12±0.01	0.10±0.00	0.85±0.08*	0.07
	34:5	752.5	0.01±0.00	0.01±0.00	1.22±0.53	0.01	0.02±0.00	0.01±0.00	**0.43±0.05***	0.01
	36:0	790.5	0.27±0.06	0.54±0.04	**1.99±0.43***	0.23	0.68±0.06	0.74±0.03	1.09±0.11	0.42
	36:1	788.5	1.97±0.24	4.07±0.23	**2.06±0.28***	1.63	6.69±0.85	8.26±0.46	1.24±0.17	4.11

	36:2	786.5	15.03±0.99	34.41±3.01	**2.29±0.25***	12.44	17.89±1.50	18.97±0.90	1.06±0.10	11.00
	36:3	784.5	9.00±1.18	16.46±1.85	**1.83±0.32***	7.45	12.13±0.84	11.08±0.65	0.91±0.08	7.43
	36:4	782.5	13.49±1.34	19.67±1.62	1.46±0.18*	11.16	14.53±0.76	11.97±0.62	0.82±0.06	8.99
	36:5	780.5	0.02±0.00	0.01±0.00	0.87±0.20	0.01	0.02±0.00	0.02±0.00	1.31±0.25	0.01
	36:6	778.5	0.08±0.01	0.11±0.01	1.42±0.20	0.06	0.10±0.01	0.09±0.00	0.86±0.08	0.06
	38:2	814.5	0.44±0.02	1.52±0.08	**3.44±0.25***	0.36	3.20±0.31	4.07±0.24	1.27±0.14*	1.97
	38:3	812.5	3.50±0.35	6.32±0.95	**1.81±0.33***	2.90	6.55±0.68	5.83±0.34	0.89±0.11	4.03
	38:4	810.5	6.75±0.49	12.89±0.97	**1.91±0.20***	5.59	9.88±0.75	8.77±0.41	0.89±0.08	6.08
	38:5	808.5	7.72±0.75	10.18±1.06	1.31±0.19	6.39	9.25±0.65	6.20±0.36	**0.65±0.06***	5.69
	38:6	806.5	12.77±1.33	20.05±1.96	**1.57±0.22**	10.57	14.66±1.15	11.05±0.60	0.75±0.07*	9.02
	40:4	838.5	0.18±0.04	0.36±0.04	**1.93±0.50***	0.15	0.63±0.07	0.51±0.03	0.81±0.10	0.39
	40:5	836.5	1.90±0.13	2.98±0.45	**1.57±0.26**	1.57	2.72±0.23	2.31±0.14	0.85±0.09	1.67
	40:6	834.5	4.60±0.27	8.64±0.70	**1.87±0.19***	3.81	6.64±0.49	5.65±0.34	0.85±0.08	4.09
	40:7	832.5	0.51±0.06	1.08±0.08	**2.12±0.29***	0.42	0.57±0.05	0.54±0.03	0.95±0.09	0.35
	40:8	830.5	0.06±0.01	0.20±0.01	**3.38±0.45***	0.05	0.11±0.01	0.11±0.00	0.94±0.11	0.07
LPE	16:0	454.5	0.03±0.01	0.04±0.00	**1.50±0.32**	10.92	0.01±0.00	0.03±0.00	**2.02±0.38**	10.47
(6)	18:0	482.5	0.02±0.00	0.02±0.00	1.36±0.34	6.23	0.02±0.00	0.06±0.00	**2.61±0.29**	19.12
	18:1	480.5	0.03±0.01	0.04±0.00	1.26±0.46	13.04	0.01±0.00	0.02±0.00	**2.05±0.37**	8.21
	18:2	478.5	0.08±0.01	0.14±0.01	**1.67±0.25**	32.43	0.03±0.00	0.03±0.00	1.28±0.17	20.00
	20:4	502.5	0.03±0.01	0.04±0.00	1.36±0.24	12.93	0.02±0.00	0.02±0.00	1.25±0.21	14.66
	22:6	526.5	0.06±0.01	0.08±0.01	1.25±0.19	24.46	0.03±0.00	0.02±0.00	**0.63±0.09**	27.54
PE	34:1	718.5	1.26±0.20	1.80±0.29	1.42±0.32	3.34	0.82±0.12	1.38±0.11	**1.67±0.29**	2.70
(16/23)	34:2	716.5	2.37±0.17	4.07±0.36	**1.72±0.20**	6.26	2.12±0.30	3.61±0.44	**1.70±0.32**	6.94
	36:1	746.5	0.87±0.16	1.06±0.13	1.22±0.27	2.29	0.47±0.07	0.77±0.12	**1.65±0.36**	1.54
	36:2	744.5	3.91±0.51	7.15±1.11	**1.83±0.37**	10.35	3.27±0.33	5.26±0.75	**1.61±0.28**	10.71
	36:3	742.5	1.61±0.09	3.43±0.21	**2.13±0.18**	4.26	1.35±0.12	2.90±0.19	**2.15±0.24**	4.43
	36:4	740.5	2.60±0.91	3.54±0.40	1.36±0.50	6.88	2.08±0.26	2.99±0.44	1.43±0.28	6.83
	38:1	774.5	0.12±0.01	0.21±0.01	**1.74±0.17**	0.32	0.12±0.02	0.14±0.01	1.17±0.23	0.41
	38:2	772.5	0.27±0.03	0.22±0.05	0.82±0.20	0.71	0.11±0.01	0.23±0.01	**1.96±0.24**	0.38
	38:3	770.5	1.46±0.27	1.89±0.26	1.29±0.30	3.87	1.01±0.06	1.86±0.15	**1.84±0.19**	3.30
	38:4	768.5	6.44±1.53	8.68±0.80	1.35±0.34	17.02	4.49±0.25	7.89±0.29	**1.75±0.12**	14.73
	38:5	766.5	2.48±0.40	3.55±0.49	1.43±0.30	6.55	2.59±0.31	2.65±0.38	1.02±0.19	8.49

	38:6	764.5	6.95±0.87	9.51±1.27	1.37±0.25	18.37	6.15±0.74	6.31±0.57	1.03±0.15	20.16
	40:4	796.5	0.28±0.04	0.28±0.04	0.99±0.21	0.74	0.14±0.02	0.22±0.02	**1.52±0.25**	0.47
	40:5	794.5	1.30±0.27	1.74±0.24	1.34±0.33	3.44	1.01±0.08	1.20±0.17	1.18±0.19	3.32
	40:6	792.5	5.33±0.96	7.14±0.69	1.34±0.27	14.10	4.33±0.58	4.99±0.62	1.15±0.21	14.21
	40:7	790.5	0.56±0.12	0.79±0.09	1.41±0.36	1.48	0.42±0.05	0.49±0.07	1.15±0.22	1.39
PEp	P-16:0/18:1	702.5	0.21±0.08	0.25±0.06	1.17±0.50	3.88	0.17±0.01	0.38±0.03	**2.17±0.23***	4.28
(9)	P-16:0/20:0	732.5	0.02±0.01	0.01±0.00	0.83±0.36	0.33	0.01±0.00	0.02±0.00	**1.94±0.39***	0.23
	P-16:0/20:2	728.5	0.01±0.00	0.02±0.01	**1.84±0.47**	0.22	0.02±0.00	0.03±0.00	1.39±0.15	0.45
	P-16:0/20:3	726.5	0.12±0.01	0.23±0.02	**1.82±0.25**	2.28	0.09±0.01	0.17±0.01	**1.88±0.33**	2.24
	P-16:0/20:4	724.5	1.42±0.17	3.21±0.31	**2.26±0.35***	26.11	1.26±0.14	2.57±0.27	**2.05±0.31***	30.98
	P-18:0/20:4	752.5	2.08±0.09	4.85±0.21	**2.33±0.14***	38.32	1.53±0.13	4.20±0.21	**2.74±0.27***	37.83
	P-18:1/20:4	750.5	1.53±0.22	2.93±0.26	**1.91±0.32**	28.16	0.94±0.04	2.51±0.18	**2.68±0.22***	23.08
	P-18:0/22:0	788.5	0.01±0.00	0.01±0.00	1.34±0.55	0.19	0.03±0.01	0.01±0.00	**0.35±0.13**	0.74
	P-18:1/22:0	786.5	0.03±0.01	0.02±0.01	0.64±0.27	0.52	0.01±0.00	0.02±0.00	**2.33±0.34***	0.17
LPA	14:0	381.5	0.24±0.05	0.11±0.02	**0.42±0.12**	4.07	0.05±0.01	0.03±0.00	**0.55±0.10**	0.59
(5)	16:0	409.5	3.18±0.18	7.34±0.36	**2.30±0.17***	54.23	4.69±0.54	1.95±0.29	**0.41±0.08***	58.62
	18:0	437.5	0.78±0.06	2.42±0.21	**3.09±0.36***	13.34	1.83±0.12	2.45±0.27	1.33±0.17	22.95
	18:1	435.5	0.77±0.12	2.01±0.17	**2.60±0.45***	13.20	0.81±0.08	1.07±0.09	1.32±0.17	10.12
	18:2	433.5	0.89±0.05	3.09±0.22	**3.47±0.32***	15.16	0.62±0.07	1.21±0.13	**1.96±0.30***	7.71
PA	16:0/18:1	673.5	0.04±0.01	0.06±0.01	**1.63±0.41**	0.86	0.10±0.03	0.19±0.02	**1.83±0.56***	1.29
(13)	16:0/18:2	671.5	0.20±0.02	0.34±0.03	**1.68±0.23***	4.92	0.51±0.06	0.17±0.07	**0.34±0.14***	6.35
	18:0/18:1	701.5	0.03±0.01	0.04±0.01	1.13±0.32	0.81	0.06±0.01	0.09±0.01	**1.54±0.36**	0.71
	18:0/18:2	699.5	0.33±0.04	0.50±0.03	**1.53±0.23**	8.01	0.91±0.1	1.04±0.07	1.14±0.15	11.30
	18:1/18:2	697.5	0.07±0.03	0.09±0.01	1.30±0.55	1.77	0.06±0.01	0.11±0.01	**1.77±0.38**	0.75
	18:2/18:2	695.5	0.07±0.02	0.10±0.02	1.41±0.44	1.69	0.15±0.03	0.12±0.01	0.81±0.19	1.89
	20:0/18:1	729.5	0.06±0.01	0.09±0.01	**1.52±0.42**	1.40	0.16±0.03	0.18±0.02	1.10±0.24	2.04
	18:0/20:4	723.5	0.06±0.01	0.04±0.01	**0.61±0.16**	1.49	0.07±0.02	0.09±0.01	**1.34±0.45**	0.83
	18:1/20:5	719.5	0.12±0.03	0.14±0.02	1.16±0.32	2.75	0.27±0.03	0.51±0.03	**1.91±0.24**	3.34
	22:0/18:2	785.5	0.13±0.01	0.25±0.03	**1.90±0.27***	3.24	0.23±0.05	0.38±0.03	**1.66±0.41**	2.81
	18:0/22:5	749.5	0.04±0.01	0.08±0.01	**2.15±0.73***	0.87	0.07±0.01	0.12±0.01	**1.78±0.33**	0.81
	18:0/22:6	747.5	0.13±0.01	0.19±0.02	1.45±0.18	3.16	0.17±0.03	0.31±0.03	**1.86±0.41***	2.07
	18:1/22:6	745.5	2.88±0.19	3.66±0.18	1.25±0.10	69.04	5.29±0.36	1.74±0.67	**0.33±0.13***	65.81
LPG	14:0	455.5	0.01±0.00	0.01±0.00	0.79±0.20	14.27	0.01±0.00	0.01±0.00	0.94±0.21	25.14

(7)	16:0	483.5	0.00±0.00	0.00±0.00	1.12±0.20	3.69	0.00±0.00	0.00±0.00	1.11±0.29	2.18
	16:1	481.5	0.00±0.00	0.00±0.00	**0.61±0.16***	2.07	0.00±0.00	0.00±0.00	0.80±0.20	0.93
	18:0	511.5	0.03±0.00	0.03±0.00	0.81±0.12*	54.52	0.03±0.00	0.03±0.00	0.91±0.05	56.85
	18:1	509.5	0.01±0.00	0.02±0.00	1.43±0.23	19.76	0.00±0.00	0.01±0.00	**1.57±0.17***	12.65
	18:2	507.5	0.00±0.00	0.00±0.00	1.17±0.17	5.41	0.00±0.00	0.00±0.00	**1.56±0.25***	1.96
	22:6	555.5	0.00±0.00	0.00±0.00	0.77±0.36	0.28	0.00±0.00	0.00±0.00	0.83±0.35	0.28
PG	16:0/16:0	721.5	0.02±0.00	0.01±0.00	0.74±0.12	1.25	0.02±0.00	0.02±0.00	0.82±0.09	1.20
(15)	16:0/16:1	719.5	0.01±0.00	0.01±0.00	0.92±0.18	0.88	0.04±0.00	0.04±0.00	1.01±0.11	2.01
	16:1/16:1	717.5	0.01±0.00	0.01±0.00	1.17±0.30	0.99	0.03±0.00	0.03±0.00	1.13±0.15	1.41
	18:1/16:0	747.5	0.02±0.00	0.01±0.00	0.87±0.13	1.33	0.02±0.00	0.02±0.00	1.03±0.15	1.05
	16:1/18:1	745.5	0.27±0.02	0.29±0.01	1.05±0.10	21.66	0.42±0.03	0.42±0.02	0.98±0.08	21.61
	16:0/18:3	743.5	0.19±0.02	0.21±0.01	1.09±0.11	15.04	0.28±0.02	0.30±0.01	1.06±0.09	14.31
	18:0/18:1	775.5	0.01±0.00	0.00±0.00	**0.65±0.11**	0.53	0.01±0.00	0.01±0.00	**0.62±0.09***	0.56
	18:1/18:1	773.5	0.09±0.01	0.10±0.00	1.07±0.11	7.26	0.17±0.02	0.18±0.02	1.08±0.15	8.70
	18:2/18:1	771.5	0.38±0.04	0.40±0.02	1.06±0.11	30.28	0.56±0.04	0.57±0.03	1.01±0.10	28.46
	18:2/18:2	769.5	0.04±0.00	0.04±0.00	0.95±0.12	3.19	0.07±0.01	0.07±0.00	0.94±0.11	3.55
	20:4/18:1	795.5	0.08±0.01	0.06±0.00	0.71±0.11	6.50	0.10±0.01	0.07±0.00	0.72±0.07	5.02
	22:6/16:0	789.5	0.03±0.00	0.02±0.00	**0.66±0.12**	2.19	0.03±0.00	0.03±0.00	0.87±0.13	1.54
	22:6/16:1	791.5	0.02±0.00	0.02±0.00	0.98±0.19	1.44	0.02±0.00	0.02±0.00	0.99±0.14	1.27
	18:1/22:6	819.5	0.01±0.00	0.01±0.00	1.04±0.18	0.78	0.01±0.00	0.01±0.00	0.84±0.14	0.71
	18:2/22:6	817.5	0.08±0.01	0.11±0.01	1.36±0.19	6.78	0.17±0.01	0.19±0.01	1.14±0.12	8.62
LPI	16:0	571.5	0.01±0.00	0.03±0.00	**2.12±0.29***	11.22	0.03±0.00	0.03±0.01	1.08±0.23	19.03
(9)	16:1	569.5	0.00±0.00	0.01±0.00	1.26±0.50	3.67	0.02±0.00	0.03±0.00	**1.53±0.19**	14.32
	18:0	599.5	0.03±0.00	0.03±0.00	1.08±0.22	23.87	0.03±0.00	0.05±0.01	1.79±0.39	19.03
	18:1	597.5	0.01±0.00	0.02±0.00	**1.83±0.39***	10.31	0.01±0.00	0.02±0.00	1.17±0.22	9.36
	18:2	595.5	0.03±0.01	0.06±0.02	**1.78±0.61**	31.35	0.01±0.00	0.02±0.01	**1.78±0.49**	5.95
	18:3	593.5	0.01±0.00	0.01±0.00	**1.87±0.97**	4.97	0.00±0.00	0.00±0.00	1.22±0.75	2.35
	20:3	621.5	0.01±0.00	0.00±0.00	0.80±0.31	4.67	0.00±0.00	0.01±0.00	1.35±0.57	2.81
	20:4	619.5	0.01±0.00	0.01±0.00	1.23±0.49	5.06	0.03±0.00	0.03±0.00	0.90±0.14	20.44

	22:6	643.5	0.01±0.00	0.01±0.00	**1.71±0.50**	4.87	0.01±0.00	0.02±0.00	**1.65±0.36**	6.69
PI	16:0/16:0	809.5	0.07±0.01	0.06±0.01	0.90±0.14	0.80	0.06±0.01	0.05±0.01	0.87±0.17	0.89
(21)	16:0/16:1	807.5	0.12±0.01	0.04±0.00	0.34±0.04*	1.38	0.02±0.00	0.01±0.00	0.82±0.17	0.26
	18:0/16:0	837.5	0.14±0.03	0.19±0.01	1.37±0.28	1.65	0.36±0.01	0.87±0.04	**2.40±0.15***	5.41
	18:1/16:0	835.5	0.41±0.11	0.44±0.04	1.09±0.31	4.88	0.62±0.02	0.96±0.09	**1.55±0.15**	9.22
	18:2/16:0	833.5	2.25±0.24	2.68±0.21	1.19±0.16	27.01	1.74±0.21	2.21±0.05	1.27±0.15	26.00
	18:2/16:1	831.5	0.05±0.00	0.04±0.01	0.80±0.16	0.63	0.08±0.02	0.08±0.01	0.91±0.27	1.24
	18:0/18:0	865.5	0.05±0.01	0.06±0.01	1.43±0.90	0.54	0.06±0.01	0.08±0.01	1.31±0.25	0.89
	18:0/18:1	863.5	0.29±0.08	0.35±0.02	1.22±0.33	3.42	0.13±0.01	0.37±0.02	**2.83±0.28***	1.94
	18:0/18:2	861.5	0.38±0.05	0.37±0.07	0.99±0.21	4.52	0.54±0.07	0.36±0.03	0.67±0.11	8.08
	18:1/18:2	859.5	0.09±0.03	0.07±0.01	0.85±0.30	1.04	0.15±0.02	0.16±0.01	1.06±0.17	2.23
	18:2/18:2	857.5	0.19±0.02	0.24±0.02	1.22±0.15	2.33	0.36±0.05	0.28±0.01	0.78±0.11	5.45
	18:2/18:3	855.5	0.08±0.01	0.13±0.02	**1.67±0.37**	0.95	0.20±0.02	0.16±0.01	0.78±0.10	3.00
	18:0/20:2	889.5	0.13±0.01	0.12±0.01	0.90±0.11	1.57	0.17±0.11	0.24±0.02	1.38±0.85	2.58
	18:0/20:3	887.5	0.11±0.01	0.09±0.01	0.79±0.13	1.33	0.07±0.01	0.11±0.01	**1.56±0.26**	1.04
	18:0/20:4	885.5	3.02±0.43	1.01±0.07	**0.33±0.05***	36.19	0.84±0.06	0.73±0.04	0.87±0.08	12.50
	18:0/20:5	883.5	0.06±0.04	0.06±0.01	0.98±0.60	0.76	0.03±0.00	0.05±0.00	**1.75±0.16***	0.41
	16:0/22:6	881.5	0.18±0.02	0.23±0.02	1.33±0.19	2.11	0.41±0.05	0.47±0.06	1.15±0.20	6.10
	18:0/22:4	913.5	0.03±0.01	0.04±0.01	1.37±0.49	0.34	0.10±0.03	0.07±0.01	0.67±0.20	1.47
	18:0/22:5	911.5	0.45±0.10	0.31±0.08	0.69±0.24	5.38	0.25±0.05	0.18±0.01	0.70±0.15	3.77
	18:0/22:6	909.5	0.25±0.05	0.21±0.02	0.83±0.18	3.05	0.48±0.06	0.41±0.03	0.85±0.12	7.20
	18:1/22:6	907.5	0.01±0.00	0.01±0.00	1.18±0.27	0.10	0.02±0.00	0.02±0.00	0.84±0.25	0.32
Sulfo HexCer	d18:1/16:0	780.5	0.31±0.06	0.27±0.02	0.90±0.19	33.94	0.27±0.03	0.38±0.02	1.41±0.19	34.55
(5)	d18:1/18:0	808.5	0.23±0.05	0.16±0.02	0.70±0.16	25.18	0.24±0.04	0.23±0.02	0.95±0.17	30.68
	d18:1/20:4	828.5	0.13±0.02	0.08±0.01	**0.62±0.12***	14.71	0.10±0.01	0.08±0.01	0.87±0.12	13.21
	d18:1/22:0	864.5	0.14±0.02	0.06±0.01	**0.44±0.08***	16.22	0.13±0.02	0.12±0.02	0.96±0.21	16.59
	d18:1/24:1	890.5	0.09±0.03	0.02±0.00	**0.27±0.08***	9.96	0.04±0.01	0.05±0.01	1.39±0.27	4.97

SM	d18:1/14:0	675.5	0.31±0.06	0.31±0.02	0.98±0.19	0.53	0.89±0.09	0.71±0.04	0.78±0.09	1.24
(18)	d18:0/16:0	705.5	0.58±0.14	0.69±0.02	1.19±0.29	0.98	1.57±0.19	1.15±0.07	0.72±0.09	2.20
	d18:1/16:0	703.5	5.15±1.04	6.29±0.20	1.22±0.25	8.71	14.98±1.34	11.93±0.46	0.80±0.07	20.83
	d18:1/16:1	701.5	0.85±0.15	1.11±0.06	1.31±0.25	1.44	1.97±0.15	1.55±0.08	0.78±0.07	2.73
	d18:1/18:0	731.5	0.94±0.06	1.29±0.06	1.35±0.10*	1.58	2.01±0.18	2.28±0.06	1.12±0.11	2.79
	d18:1/18:1	729.5	0.60±0.05	0.78±0.03	1.31±0.11	1.01	0.96±0.08	1.00±0.03	1.04±0.10	1.32
	d18:1/20:0	759.5	19.40±0.77	24.75±0.76	1.28±0.06*	32.91	17.11±1.74	12.40±0.51	0.72±0.08*	23.71
	d18:1/20:2	755.5	0.08±0.02	0.06±0.01	0.81±0.26	0.13	0.07±0.01	0.05±0.00	0.70±0.10	0.09
	d18:1/20:6	747.5	0.15±0.01	0.24±0.03	**1.54±0.22***	0.26	0.23±0.02	0.26±0.02	1.13±0.14	0.32
	d18:1/22:0	787.5	9.89±0.45	16.17±0.73	**1.63±0.10***	16.55	9.99±1.72	8.66±0.32	0.86±0.16	13.75
	d18:1/22:1	785.5	8.58±1.01	9.96±0.21	1.16±0.14	14.52	7.00±0.54	5.63±0.12	0.81±0.07	9.53
	d18:1/22:6	775.5	0.20±0.02	0.27±0.02	1.34±0.22	0.33	0.20±0.01	0.25±0.01	1.27±0.09	0.27
	d18:1/23:0	801.5	0.45±0.05	0.55±0.06	1.22±0.18	0.77	1.04±0.24	0.93±0.11	0.88±0.23	1.45
	d18:0/24:0	817.5	0.11±0.01	0.12±0.02	1.11±0.21	0.18	0.14±0.01	0.26±0.00	1.86±0.09*	0.19
	d18:1/24:0	815.5	1.01±0.14	0.97±0.13	0.96±0.18	1.75	1.23±0.27	1.18±0.21	0.96±0.26	1.74
	d18:1/24:1	813.5	3.33±0.46	4.67±0.54	1.40±0.25	5.69	7.11±2.43	6.31±0.45	0.88±0.32	9.83
	d18:1/24:2	811.5	7.47±0.37	8.64±0.36	1.14±0.07	12.64	5.78±0.47	4.10±0.14	0.70±0.08*	7.98
	d20:1/24:0	843.5	0.03±0.00	0.01±0.00	**0.56±0.07**	0.04	0.03±0.00	0.01±0.00	**0.43±0.05**	0.04
Cer	d18:1/16:0	538.5	0.05±0.02	0.03±0.00	**0.59±0.21**	7.79	0.10±0.01	0.41±0.03	**4.10±0.43***	5.06
(12)	d18:1/16:1	536.5	0.15±0.06	0.03±0.00	**0.19±0.08***	21.64	0.05±0.00	0.03±0.00	**0.62±0.06***	2.68
	d18:1/18:0	566.5	0.03±0.01	0.01±0.00	**0.49±0.27**	3.53	0.04±0.00	0.13±0.01	**3.36±0.51**	1.98
	d18:1/18:1	564.5	0.01±0.00	0.00±0.00	**0.30±0.10**	1.71	0.01±0.00	0.01±0.00	1.36±0.20	0.35
	d18:1/20:2	590.5	0.03±0.01	0.01±0.00	**0.19±0.09***	3.80	0.01±0.00	0.02±0.00	**1.56±0.34**	0.73
	d18:1/22:0	622.5	0.04±0.01	0.06±0.00	**1.50±0.25**	6.07	0.28±0.03	0.53±0.08	**1.91±0.34**	14.09
	d18:1/22:1	620.5	0.04±0.03	0.01±0.00	**0.19±0.15***	5.89	0.02±0.00	0.03±0.01	1.40±0.51	1.23
	d18:1/22:6	610.5	0.01±0.00	0.00±0.00	**0.34±0.16**	1.29	0.00±0.00	0.01±0.00	**2.16±0.50**	0.27
	d18:0/24:0	652.5	0.01±0.00	0.01±0.00	0.89±0.23	1.28	0.05±0.01	0.07±0.02	1.49±0.51	2.44
	d18:1/24:0	650.5	0.16±0.03	0.21±0.02	1.35±0.28	22.12	1.02±0.20	1.79±0.10	**1.75±0.35**	51.31

	d18:1/24:1	648.5	0.16±0.01	0.11±0.01	0.68±0.09	22.78	0.38±0.03	0.69±0.08	**1.82±0.25**	18.94
	d20:1/24:0	678.5	0.02±0.00	0.01±0.00	**0.50±0.13**	2.10	0.02±0.00	0.03±0.00	**1.65±0.45**	0.93
HexCer	d18:1/16:0	700.5	0.20±0.01	0.54±0.02	**2.66±0.15***	7.81	0.32±0.02	0.50±0.03	**1.58±0.13**	6.03
(11)	d18:1/16:1	698.5	0.08±0.00	0.18±0.01	**2.31±0.20***	3.00	1.92±0.17	0.38±0.01	**0.19±0.02***	36.49
	d18:1/18:0	728.5	0.07±0.01	0.12±0.01	**1.70±0.33***	2.74	0.40±0.06	0.12±0.01	**0.30±0.05***	7.67
	d18:1/18:1	726.5	0.05±0.01	0.09±0.01	**1.85±0.44***	1.98	0.39±0.04	0.18±0.00	**0.46±0.05***	7.38
	d18:1/20:0	756.5	0.24±0.04	0.33±0.04	1.40±0.26	9.14	0.05±0.00	0.08±0.00	**1.71±0.13**	0.90
	d18:1/20:1	754.5	0.07±0.01	0.10±0.01	**1.51±0.33***	2.52	0.03±0.00	0.03±0.00	1.02±0.16	0.60
	d18:1/22:0	784.5	0.40±0.07	0.17±0.01	**0.42±0.08***	15.51	0.43±0.03	1.00±0.07	**2.30±0.21***	8.24
	d18:1/22:1	782.5	0.12±0.03	0.06±0.01	**0.52±0.15***	4.56	0.23±0.03	0.15±0.01	**0.64±0.10**	4.38
	d18:1/24:0	812.5	0.50±0.07	0.25±0.02	**0.50±0.08***	19.34	0.59±0.05	1.11±0.06	**1.88±0.18***	11.21
	d18:1/24:1	810.5	0.63±0.10	0.21±0.01	**0.33±0.06***	24.33	0.51±0.03	1.15±0.06	**2.26±0.17***	9.70
	d18:1/24:2	808.5	0.23±0.02	0.81±0.02	**3.48±0.36***	9.05	0.39±0.04	0.39±0.02	1.01±0.12	7.39
Hex2Cer	d18:1/16:0	862.5	0.26±0.17	0.22±0.06	0.83±0.60	21.27	0.95±0.07	1.45±0.05	**1.52±0.12**	70.00
(3)	d18:1/24:0	974.5	0.48±0.03	0.54±0.09	1.13±0.19	38.60	0.12±0.01	0.13±0.01	1.12±0.16	8.49
	d18:1/24:1	972.5	0.49±0.05	0.36±0.02	0.72±0.09	40.12	0.29±0.03	0.45±0.22	**1.54±0.76**	21.56
CL	$(18:1)_4$	727.5	0.08±0.01	0.09±0.01	1.12±0.22	18.11	0.18±0.03	0.20±0.04	1.12±0.26	21.62
(3)	$(18:1, 18:2)_4$	725.5	0.35±0.05	0.40±0.04	1.15±0.20	74.58	0.59±0.06	0.44±0.06	0.74±0.12	72.04
	$(18:2)_4$	723.5	0.03±0.01	0.07±0.01	**2.00±0.52**	7.30	0.05±0.01	0.08±0.01	**1.65±0.36**	6.34
DG	14:1_16:0	556.5	2.06±0.35	1.47±0.20	0.71±0.15	3.35	2.42±0.44	1.32±0.19	**0.54±0.13**	2.09
(18)	16:0_16:0	586.5	10.70±2.00	9.92±1.11	0.92±0.20	17.36	12.77±1.60	21.61±3.12	**1.69±0.32**	11.00
	16:0_18:0	614.5	2.47±1.06	1.86±0.21	0.75±0.33	4.01	2.10±0.25	3.08±0.36	1.46±0.24	1.81
	16:0_18:1	612.5	0.90±0.20	0.58±0.05	**0.65±0.16**	1.45	1.03±0.08	0.91±0.09	0.86±0.11	0.89
	16:1_18:1	610.5	6.12±0.76	8.00±0.85	1.29±0.21	9.93	14.50±1.51	40.23±6.64	**2.66±0.54**	12.49
	16:1_18:3	606.5	0.20±0.05	0.13±0.02	**0.66±0.18**	0.32	0.15±0.03	0.10±0.01	**0.65±0.18**	0.13
	18:0_18:0	642.5	0.87±0.18	0.85±0.11	0.97±0.24	1.41	0.59±0.07	1.02±0.07	**1.79±0.23**	0.50
	18:0_18:1	640.5	2.43±0.30	2.26±0.28	0.93±0.16	3.94	3.35±0.38	6.74±0.79	**2.00±0.32**	2.88
	18:0_18:2	638.5	18.68±2.04	21.55±1.80	1.15±0.16	30.33	38.67±4.68	68.24±9.37	**1.75±0.32**	33.31
	18:1_18:2	636.5	6.83±0.55	7.82±0.63	1.15±0.13	11.08	21.84±3.37	31.76±3.20	1.45±0.27	21.85
	18:2_18:2	634.5	3.83±0.57	2.71±0.28	0.70±0.12	6.23	7.25±1.11	7.77±0.75	1.01±0.19	6.25
	16:1_22:1	666.5	0.49±0.13	0.67±0.09	1.30±0.40	0.80	0.34±0.09	0.40±0.04	1.14±0.36	0.29
	18:2_20:2	662.5	0.23±0.06	0.43±0.08	**1.88±0.59**	0.37	0.76±0.20	2.06±0.13	**2.72±0.75***	0.65

	20:0_20:0	698.5	2.72±0.96	1.03±0.13	**0.37±0.14**	4.42	1.29±0.22	1.08±0.13	0.84±0.18	1.11
	20:0_20:1	696.5	0.10±0.03	0.08±0.02	0.87±0.36	0.16	0.35±0.13	0.81±0.07	**2.34±0.91**	0.30
	20:0_20:3	692.5	0.34±0.14	0.58±0.08	**1.71±0.75**	0.55	0.45±0.15	0.21±0.03	**0.43±0.15**	0.39
	20:0_20:5	688.5	1.53±0.35	1.33±0.30	0.86±0.28	2.49	7.29±2.88	3.40±0.49	**0.46±0.19**	6.28
	18:0_22:6	686.5	1.12±0.29	1.03±0.11	0.92±0.26	1.81	0.95±0.07	2.19±0.15	**2.31±0.24**	0.82
TG	42:0	740.8	0.96±0.13	1.62±0.31	**1.69±0.39**	3.39	0.32±0.05	0.53±0.07	**1.69±0.35**	3.04
(41/141)	42:1	738.8	0.00±0.00	0.00±0.00	**0.51±0.21**	0.01	0.00±0.00	0.00±0.00	**1.58±0.63**	0.03
	42:2	736.8	0.00±0.00	0.00±0.00	**1.67±0.29**	0.01	0.00±0.00	0.01±0.00	**1.57±0.22**	0.04
	44:2	764.8	0.00±0.00	0.03±0.00	**3.97±0.36***	0.03	0.01±0.00	0.02±0.00	**1.71±0.40**	0.01
	46:0	796.8	0.44±0.04	0.31±0.08	0.70±0.18	1.56	0.25±0.05	0.33±0.06	1.32±0.36	2.41
	46:1	794.8	0.31±0.03	0.25±0.03	0.79±0.12	1.10	0.25±0.03	0.45±0.06	**1.76±0.29**	2.44
	46:2	792.8	0.45±0.06	0.40±0.11	0.90±0.27	1.51	0.32±0.07	0.38±0.11	1.18±0.43	3.08
	46:3	790.8	0.04±0.01	0.04±0.01	0.86±0.35	0.15	0.03±0.01	0.03±0.01	1.03±0.43	0.31
	46:4	788.8	0.00±0.00	0.00±0.00	1.41±0.92	0.01	0.00±0.00	0.00±0.00	0.78±0.65	0.02
	48:1	822.8	0.83±0.14	0.53±0.12	**0.64±0.18**	2.92	0.57±0.10	0.54±0.10	0.96±0.24	5.45
	48:2	820.8	1.22±0.13	1.69±0.49	1.38±0.43	4.30	0.81±0.17	0.98±0.22	1.21±0.37	7.80
	48:3	818.8	0.41±0.05	0.72±0.16	**1.73±0.44**	1.45	0.22±0.02	0.36±0.06	**1.60±0.27**	2.16
	48:4	816.8	0.29±0.04	0.24±0.05	0.84±0.19	1.02	0.09±0.01	0.20±0.01	**2.16±0.21***	0.91
	48:5	814.8	0.03±0.00	0.03±0.01	1.05±0.24	0.10	0.01±0.00	0.02±0.00	**1.82±0.38**	0.09
	50:0	852.8	0.76±0.11	0.57±0.15	0.75±0.23	2.66	0.46±0.10	0.64±0.10	1.40±0.38	4.40
	50:1	850.8	2.72±0.31	2.58±0.69	0.95±0.28	9.57	0.65±0.09	1.06±0.11	**1.64±0.28**	6.25
	50:2	848.8	1.55±0.14	1.75±0.24	1.13±0.19	5.44	0.65±0.11	1.20±0.09	**1.85±0.35**	6.25
	50:3	846.8	0.18±0.00	0.30±0.02	**1.62±0.09**	0.64	0.27±0.02	0.56±0.02	**2.07±0.17**	2.62
	50:4	844.8	1.09±0.12	1.51±0.31	1.39±0.32	3.82	0.55±0.10	0.94±0.18	**1.69±0.45***	5.34
	50:5	842.8	0.00±0.00	0.00±0.00	0.75±0.35	0.01	0.01±0.00	0.02±0.00	1.42±0.19	0.13
	50:6	840.8	0.00±0.00	0.00±0.00	1.48±0.73	0.01	0.00±0.00	0.01±0.00	**1.55±0.82**	0.03
	52:0	880.8	0.26±0.05	0.13±0.02	**0.51±0.14***	0.91	0.18±0.03	0.23±0.06	1.25±0.41	1.76
	52:1	878.8	0.35±0.05	0.56±0.13	**1.58±0.43**	1.24	0.15±0.03	0.22±0.02	1.48±0.32	1.43
	52:2	876.8	4.71±0.49	5.08±1.10	1.08±0.26	16.55	1.24±0.11	2.33±0.18	**1.87±0.22**	12.00
	52:3	874.8	3.77±0.47	3.57±0.59	0.95±0.20	13.27	0.94±0.07	1.49±0.10	**1.59±0.16**	9.04
	52:4	872.8	2.90±0.36	2.44±0.46	0.84±0.19	10.21	0.76±0.02	1.90±0.07	**2.50±0.12**	7.33
	52:5	870.8	0.24±0.03	0.27±0.05	1.12±0.25	0.85	0.11±0.01	0.31±0.03	**2.93±0.33**	1.01
	52:6	868.8	0.01±0.00	0.01±0.00	0.91±0.38	0.04	0.01±0.00	0.01±0.00	**1.51±0.19**	0.08

52:7	866.8	0.02±0.00	0.02±0.01	1.05±0.44	0.08	0.02±0.00	0.02±0.01	1.12±0.41	0.16
54:1	906.8	0.36±0.04	0.35±0.07	0.96±0.21	1.28	0.12±0.02	0.22±0.02	**1.88±0.32**	1.14
54:2	904.8	0.24±0.04	0.16±0.05	0.68±0.23	0.83	0.06±0.02	0.07±0.01	1.13±0.36	0.61
54:3	902.8	0.08±0.02	0.09±0.02	1.10±0.33	0.28	0.09±0.01	0.19±0.01	**1.98±0.20***	0.90
54:4	900.8	1.38±0.29	1.68±0.37	1.22±0.37	<u>4.86</u>	0.35±0.04	0.52±0.05	1.50±0.22	<u>3.34</u>
54:5	898.8	0.55±0.02	1.46±0.07	**2.65±0.15***	1.94	0.27±0.02	0.40±0.02	**1.51±0.14**	<u>2.57</u>
54:6	896.8	1.58±0.21	1.08±0.31	0.68±0.22	<u>5.55</u>	0.34±0.02	1.05±0.08	**3.12±0.29***	<u>3.24</u>
54:7	894.8	0.15±0.02	0.15±0.03	1.05±0.24	0.51	0.05±0.01	0.09±0.01	**1.81±0.31**	0.46
56:6	924.8	0.01±0.00	0.03±0.00	**3.37±0.18***	0.04	0.05±0.00	0.10±0.01	**2.19±0.21**	0.45
56:9	918.8	0.12±0.01	0.22±0.04	**1.91±0.43***	0.41	0.04±0.00	0.09±0.00	**2.30±0.27***	0.36
56:10	916.8	0.12±0.01	0.19±0.04	**1.55±0.35**	0.43	0.04±0.00	0.09±0.00	**2.22±0.27***	0.38
58:9	946.8	0.08±0.01	0.08±0.01	0.96±0.24	0.28	0.02±0.00	0.05±0.00	**1.93±0.34***	0.23
60:13	986.8	0.19±0.02	0.19±0.04	0.97±0.22	0.68	0.06±0.00	0.16±0.01	**2.52±0.24**	0.61

Table S5. Potentially available acyl chains of a) PC, b) PE and c) TG based on CID spectra using nUHPLC-ESI-MS/MS.

a) PC

molecular species	possible acyl chains	*m/z*	molecular species	possible acyl chains	*m/z*	molecular species	possible acyl chains	*m/z*
28:2	14:0/14:2	674.5		18:0/18:1			16:1/22:4	
30:0	14:0/16:0	706.5	36:2	18:0/18:2	786.5		18:1/20:4	
32:0	16:0/16:0	734.5		18:1/18:1			18:0/20:5	
32:1	14:0/18:1	732.5	36:3	16:0/20:3	784.5		20:3/18:2	
	16:0/16:1			18:1/18:2		38:6	18:1/20:5	806.5
32:2	16:1/16:1	730.5	36:4	16:0/20:4	782.5		18:2/20:4	
	14:0/18:2		36:5	16:0/20:5	780.5		16:1/22:5	
34:0	18:0/16:0	762.5	36:6	16:1/20:5	778.5		16:0/22:6	
34:1	16:0/18:1	760.5	38:2	18:0/20:2	814.5	40:4	16:0/24:4	838.5
34:2	16:0/18:2	758.5		20:0/18:2			18:0/22:4	
34:3	16:0/18:3	756.5	38:3	18:0/20:3	812.5	40:5	18:0/22:5	836.5
34:4	14:0/20:4	754.5		18:1/20:2			18:1/22:4	
34:5	14:0/20:5	752.5	38:4	16:0/22:4	810.5		20:1/20:4	
36:0	16:0/20:0	790.5		20:3/18:1		40:6	18:0/22:6	834.5
	18:0/18:0			20:4/18:0		40:7	18:1/22:6	832.5
36:1	16:0/20:1	788.5	38:5	16:0/22:5	808.5	40:8	18:2/22:6	830.5

b) PE

molecular species	possible acyl chains	*m/z*	molecular species	possible acyl chains	*m/z*
34:1	18:1/16:0	718.5		18:1/20:2	
34:2	16:0/18:2	716.5	38:4	16:0/22:4	768.5
36:1	20:1/16:0	746.5		20:4/18:0	
	18:0/18:1		38:5	16:0/22:5	766.5
36:2	18:1/18:1	744.5		16:1/22:4	
36:3	16:0/20:3	742.5	38:6	18:0/20:6	764.5
	18:1/18:2		40:4	18:0/22:4	796.5
36:4	16:0/20:4	740.5	40:5	18:0/22:5	794.5
38:1	18:0/20:1	774.5		20:5/20:0	
	18:1/20:0		40:6	18:0/22:6	792.5
38:2	20:0/18:2	772.5	40:7	18:1/22:6	790.5
38:3	18:0/20:3	770.5			

c) TG

molecular species	possible acyl chains	*m/z*	molecular species	possible acyl chains	*m/z*	molecular species	possible acyl chains	*m/z*
42:0	14:0_14:0_14:0	740.8		14:0_16:1_18:3			16:0_18:2_18:2	
42:1	14:0_14:0_14:1	738.8		14:0_16:2_18:2			16:1_18:0_18:3	
42:2	14:0_14:1_14:1	736.8		14:1_16:1_18:2			16:1_18:1_18:2	
44:2	12:0_14:0_18:2	764.8		16:1_16:1_16:2		52:5	16:0_16:1_20:4	870.8
	12:0_14:1_18:1		48:5	12:0_18:2_18:3	814.8		16:0_18:1_18:4	
	12:1_14:0_18:1			12:0_18:1_18:4			16:0_18:2_18:3	
	12:0_16:0_16:2			14:0_16:1_18:4			16:1_18:2_18:2	
	12:0_16:1_16:1			14:1_16:0_18:4			16:1_18:1_18:3	
	14:0_14:1_16:1		50:0	14:0_18:0_18:0	852.8	52:6	14:0_18:2_20:4	868.8
	12:1_16:1_16:0		50:1	14:0_18:0_18:1	850.8		16:1_16:1_20:4	
46:0	14:0_16:0_16:0	796.8		14:0_16:0_20:1			16:0_16:1_20:5	
46:1	14:0_14:0_18:1	794.8		16:0_16:0_18:1			16:0_16:2_20:4	
	12:0_16:0_18:1			16:0_16:1_18:0			16:0_18:3_18:3	
	12:0_16:1_18:0		50:2	16:0_16:1_18:1	848.8	52:7	14:1_16:0_22:6	866.8
	14:0_16:0_16:1			16:0_16:0_18:2			14:0_16:1_22:6	
	14:1_16:0_16:0			14:0_18:1_18:1		54:1	16:0_18:0_20:1	906.8
46:2	12:0_16:1_18:1	792.8	50:3	14:0_18:1_18:2	846.8		16:0_18:1_20:0	
	12:0_16:0_18:2			14:0_16:0_20:3			18:0_18:0_18:1	
	12:1_16:0_18:1			16:0_16:1_18:2		54:2	16:0_18:2_20:0	904.8
	14:0_14:0_18:2		50:4	14:0_18:1_18:3	844.8		16:0_18:1_20:1	
	14:0_14:1_18:1			14:0_18:2_18:2		54:3	16:0_18:1_20:2	902.8
46:3	12:0_16:0_18:3	790.8		16:0_16:0_18:4			18:1_18:1_18:1	
	12:0_16:1_18:2			16:1_16:0_18:3			18:0_18:1_18:2	
	12:0_16:2_18:1			16:0_16:2_18:2		54:4	16:0_18:1_20:3	900.8
	12:1_16:1_18:1			14:1_18:0_18:3			18:0_18:1_18:3	
	12:1_16:0_18:2		50:5	14:1_18:2_18:2	842.8		18:0_18:2_18:2	
	14:0_14:1_18:2			16:1_16:1_18:3		54:5	16:0_18:1_20:4	898.8
	14:1_14:1_18:1			16:1_16:2_18:2			16:1_18:0_20:4	
46:4	12:0_16:0_18:4	788.8	50:6	14:0_18:2_18:4	840.8		16:0_18:2_20:3	
	14:0_16:1_16:3			14:0_16:1_20:5		54:6	16:0_18:1_20:5	896.8
	12:0_16:3_18:0			14:1_16:1_20:4			16:1_18:1_20:4	
	12:0_14:0_20:4			14:1_18:1_18:4		54:7	16:0_16:1_22:6	894.8
48:1	14:0_16:0_18:1	822.8		14:0_18:3_18:3			16:0_18:1_20:5	
	14:0_16:1_18:0			14:2_18:1_18:3			14:0_18:1_22:6	
	16:0_16:0_16:1		52:0	16:0_16:0_20:0	880.7	56:6	16:0_180_22:6	924.8
48:2	12:0_18:1_18:1	820.8		16:0_18:0_18:0			16:0_18:1_22:5	
	14:0_16:1_18:1		52:1	16:0_16:1_20:0	878.8		16:0_18:2_22:4	
	14:1_16:0_18:1			16:1_18:0_18:0			16:1_18:2_20:3	
	14:0_16:0_18:2			14:0_18:0_20:1		56:9	16:0_18:3_22:6	918.8
	16:0_16:1_16:1			16:0_18:0_18:1			16:1_18:2_22:6	
48:3	12:0_18:2_18:2	818.8	52:2	16:0_18:0_18:2	876.8		16:0_20:4_20:5	

	14:0_16:1_18:2			16:0_18:1_18:1		56:10	16:1_18:3_22:6	916.8
	14:0_16:2_18:1			16:1_18:0_18:1			16:1_20:4_20:5	
	14:0_16:0_18:3		52:3	16:0_16:1_20:2	874.8	58:9	18:1_18:2_22:6	946.8
	14:1_16:0_18:2			16:0_18:1_18:2		60:13	18:2_20:5_22:6	966.8
	16:1_16:1_16:1			16:0_16:0_20:3				
48:4	12:0_18:2_18:2	816.8		16:1_18:1_18:1				
	12:0_18:1_18:3		52:4	16:0_18:1_18:3	872.8			

Table S6. Relative ratio (PMOp/control) of lipid amounts in each lipoprotein fraction between PMOp and control groups selected for lipid species showing significant changes (> 1.5-fold and p-value < 0.05, marked with *) either in HDL or LDL fraction of patients. Number percentage in "abun" represents the relative abundance in each lipid class.

class	molecular species	*m/z*	HDL				LDL			
			control (n=10)	PMOp (n=10)	PMOp/control	abun. (%)	control (n=10)	PMOp (n=10)	PMOp/control	abun. (%)
PC	34:1	760.5	12.14±0.88	27.61±1.08	**2.28±0.19***	10.04	18.12±1.66	19.27±0.96	1.06±0.11	11.15
	34:2	758.5	25.60±3.67	46.10±4.31	**1.80±0.31***	21.18	26.47±2.00	22.79±1.11	0.86±0.08	16.28
	36:2	786.5	15.03±0.99	34.41±3.01	**2.29±0.25***	12.44	17.89±1.50	18.97±0.90	1.06±0.10	11.00
	36:3	784.5	9.00±1.18	16.46±1.85	**1.83±0.32***	7.45	12.13±0.84	11.08±0.65	0.91±0.08	7.43
	38:4	810.5	6.75±0.49	12.89±0.97	**1.91±0.20***	5.59	9.88±0.75	8.77±0.41	0.89±0.08	6.08
	38:5	808.5	7.72±0.75	10.18±1.06	1.31±0.19	6.39	9.25±0.65	6.20±0.36	**0.65±0.06***	5.69
	40:6	834.5	4.60±0.27	8.64±0.70	**1.87±0.19***	3.81	6.64±0.49	5.65±0.34	0.85±0.08	4.09
PEp	P-16:0/20:4	724.5	1.42±0.17	3.21±0.31	**2.26±0.35***	26.11	1.26±0.14	2.57±0.27	**2.05±0.31***	30.98
	P-18:0/20:4	752.5	2.08±0.09	4.85±0.21	**2.33±0.14***	38.32	1.53±0.13	4.20±0.21	**2.74±0.27***	37.83
	P-18:1/20:4	750.5	1.53±0.22	2.93±0.26	**1.91±0.32**	28.16	0.94±0.04	2.51±0.18	**2.68±0.22***	23.08
LPA	16:0	409.5	3.18±0.18	7.34±0.36	**2.30±0.17***	54.23	4.69±0.54	1.95±0.29	**0.41±0.08***	58.62
PA	18:1/22:6	745.5	2.88±0.19	3.66±0.18	1.25±0.10	69.04	5.29±0.36	1.74±0.67	**0.33±0.13***	65.81
LPI	16:0	571.5	0.01±0.00	0.03±0.00	**2.12±0.29***	11.22	0.03±0.00	0.03±0.01	1.08±0.23	19.03
PI	18:0/20:4	885.5	3.02±0.43	1.01±0.07	**0.33±0.05***	36.19	0.84±0.06	0.73±0.04	0.87±0.08	12.50
SM	d18:1/22:0	787.5	9.89±0.45	16.17±0.73	**1.63±0.10***	16.55	9.99±1.72	8.66±0.32	0.86±0.16	13.75
Cer	d18:1/16:1	536.5	0.15±0.06	0.03±0.00	**0.19±0.08***	21.64	0.05±0.00	0.03±0.00	**0.62±0.06***	2.68
HexCer	d18:1/16:1	698.5	0.08±0.00	0.18±0.01	**2.31±0.20***	3.00	1.92±0.17	0.38±0.01	**0.19±0.02***	36.49
	d18:1/22:0	784.5	0.40±0.07	0.17±0.01	**0.42±0.08***	15.51	0.43±0.03	1.00±0.07	**2.30±0.21***	8.24
	d18:1/24:0	812.5	0.50±0.07	0.25±0.02	**0.50±0.08***	19.34	0.59±0.05	1.11±0.06	**1.88±0.18***	11.21
	d18:1/24:1	810.5	0.63±0.10	0.21±0.01	**0.33±0.06***	24.33	0.51±0.03	1.15±0.06	**2.26±0.17***	9.70
TG	50:4	844.8	1.09±0.12	1.51±0.31	1.39±0.32	3.82	0.55±0.10	0.94±0.18	**1.69±0.45***	5.34
	54:6	896.8	1.58±0.21	1.08±0.31	0.68±0.22	5.55	0.34±0.02	1.05±0.08	**3.12±0.29***	3.24
